# Effects of Electro-Acupuncture on Ovarian P450arom, P450c17α and mRNA Expression Induced by Letrozole in PCOS Rats

**DOI:** 10.1371/journal.pone.0079382

**Published:** 2013-11-18

**Authors:** Jie Sun, Chunlan Jin, Huangan Wu, Jimeng Zhao, Yunhua Cui, Huirong Liu, Lingxiang Wu, Yin Shi, Bing Zhu

**Affiliations:** 1 Shanghai University of TCM, Shanghai, China; 2 Shanghai Institute of Acupuncture-Moxibustion and Meridians, Shanghai, China; 3 Institute of Acu-Moxibustion, China Academy of Chinese Medical Sciences, Beijing, China; Clermont Université, France

## Abstract

Hyperandrogenism is a core factor in the series of reproductive and endocrine metabolic disorders involved in polycystic ovary syndrome (PCOS). Abnormalities in enzymatic activity and the expression of ovarian granular cell layer P450arom and theca cell P450c17α can lead to an atypical environment of local ovarian hormones, including excessive androgen levels. Rat models prepared with letrozole exhibit similar endocrine and histological changes to those that occur in human PCOS. We used such a model to study the role of electro-acupuncture (EA) in regulating ovarian P450arom and P450c17α enzymatic activity and mRNA expression in PCOS rats. Female Sprague Dawley (SD) rats aged 42 days were randomly divided into 3 groups (control, PCOS, and PCOS EA) consisting of 10 rats each. The PCOS and PCOS EA groups were administered a gavage of 1.0 mg/kg^−1^ of letrozole solution once daily for 21 consecutive days. Beginning in the ninth week, the PCOS EA group was administered low-frequency EA treatment daily for 14 consecutive days. After the treatment, we obtained the following results. The estrous cycles were restored in 8 of the 10 rats in the PCOS EA group, and their ovarian morphologies and ultrastructures normalized. The peripheral blood measurements (with ELISA) showed significantly decreased androgens (i.e., androstenedione and testosterone) with significantly increased estrogens (i.e., estrone, estradiol) and increased P450arom with decreased P450C17α. Immunohistochemistry and Western blotting methods showed enhanced expression of ovarian granular cell layer P450arom as well as decreased expression of theca cell layer P450C17α. Fluorescence quantitative PCR methods showed enhanced expression of ovarian granular cell layer P450arom mRNA as well as decreased expression of theca cell layer P450C17α mRNA. These results may help explain the effects of electro-acupuncture in changing the local ovarian hyperandrogenic environment and improving reproductive and endocrine metabolic disorders in PCOS.

## Introduction

Polycystic ovary syndrome (PCOS) is a female reproductive and endocrine disease that results in follicular development and ovulation disorders because of steroid hormone imbalances. The basic clinical and pathological features of PCOS are chronic persistent anovulation and hyperandrogenism, bilateral cystic enlargement of the ovaries (larger than normal ovaries in all follicle periods, including preantral), and significantly increased androgen levels with relatively insufficient estradiol in the peripheral blood [1]. Studies have found that increased androgen levels, or hyperandrogenism, constitute a core feature of PCOS-related reproductive and endocrine metabolic disorders [2]. Local ovarian hyperandrogenism, which is caused by abnormalities in enzymatic activity or in the expression of ovarian granular cell layer P450arom and theca cell P450c17α, plays a vital role in local ovarian endocrine disorders such as PCOS [3]–[4].

In recent years, electro-acupuncture (EA) has shown advantages in treating PCOS through multi-channel and multi-targeted regulation of the reproductive and endocrine functions along the hypothalamic-pituitary-ovarian axis [5]–[10]. EA can normalize a patient’s ovulation and menstrual cycles by affecting or improving hypothalamus, pituitary, and ovarian functions [11]–[13]. We have previously reported that acupuncture could significantly reduce the weights, body mass indices (BMIs), and waist-hip ratios (WHRs) of obese patients with PCOS; decrease their peripheral testosterone levels and the ratio of luteinizing hormone (LH) to follicle stimulating hormone (FSH); and improve insulin resistance and menstrual cycle and ovulation dysfunction [14].

This study was undertaken to observe whether EA would affect the expression of P450arom and P450c17α and their mRNA expression in PCOS ovarian tissues using letrozole, a non-steroidal aromatase inhibitor, as a reference for preparing a PCOS animal model (similar to that of Kafali [15]). In this study, we accounted for the following factors: the PCOS ovarian tissue morphology and structural changes, ovarian expression of P450arom, P450c17α and their mRNA, and steroid hormones (i.e., testosterone, androstenedione, estradiol, and estrone) levels related to the synthesis or catalysis of the 2 enzymes.

## Materials and Methods

### Experimental Animal and Ethics Statement

We purchased 6-week-old specific-pathogen free (SPF) level inbred female Sprague Dawley (SD) rats, weighting 200 g±20 g, from the Experimental Animal Science Department of Fudan University. All animal experiments were approved by the Fudan University Committee on Laboratory Animals (protocol number ETCA2013BN0001 and EA20130001B), and the experiments were performed in strict accordance with the guidelines of the Chinese Council on Animal Care. All rats were provided with humane care in a temperature-controlled room with a 12-hr light-dark cycle and ad libitum access to food and water in their cages.

### Study Procedure

The 30 (42-day-old) female SD rats were randomly assigned into 3 groups of 10 rats each: the control, PCOS, and PCOS EA groups. The PCOS and PCOS EA groups were administered a gavage of 1.0 mg/kg^−1^ of letrozole solution once daily for 21 consecutive days. The control group was administered a gavage of normal saline. From the first day of modeling (i.e., the 43rd day), vaginal smears were performed on each group, and their estrous cycles were observed for 36 consecutive days. From the second day after modeling (i.e., the 64th day), the PCOS EA group was administered EA treatment for 14 consecutive days. From the second day after the EA treatment (i.e., the 78th day), peripheral blood and ovarian tissue samples were collected from each group. The rats were anesthetized with 2% pentobarbital sodium (100 µg/g of body weight), and then blood samples were obtained directly from the heart. The blood samples were centrifuged (3000×g for 10 min at 4°C), and the serum was stored at 20°C until extraction. The ovaries were quickly removed and dissected on dry ice; they were then stored at −80°C until extraction.

### EA Treatment

In the PCOS EA group, acupoints CV-4 (on the ventral midline at approximately the upper 4/5 and lower 1/5 of the line joining the xiphoid and pubic symphysis with the T12 to L2 abdominal nerves and spinal nerves under the skin) and CV-3 were used. During the operation, Φ0.22×13 mm acupuncture needles were quickly inserted 2–3 mm into the acupoints; the needles were then connected to a HANS-100 pain treatment device (Nanjing Jisheng Medical Technology Co., Ltd, Nanjing, China), which uses a continuous wave 2 Hz frequency and a 2 mA current, until quivering of the acupoint area was visible. Needle retention lasted 20 min. Treatment was administered once daily for 14 consecutive days.

### Vaginal Smears

Vaginal smears were performed on all of the rats daily at 9∶00 am beginning with the 43rd day, and their estrous cycles were observed. The observation period lasted for 36 consecutive days. A sterile cotton swab was soaked in 0.9% saline before it was smeared around the first 1/3 of the vaginal wall. The cotton swab was removed and was smeared in the same direction on a glass slide. The smear containing vaginal cells was fixed in absolute ethanol for 8 min and then stained with Wright’s stain (Beijing Cell Chip Biotechnology Co., Ltd, Beijing, China), with drops placed on the stain until the entire smear was covered. After 30 s to 1 min, an equal amount of phosphate buffer was applied (pH 6.4∼6.8). The slides were stained for 3 to 5 min before being gradually washed with tap water. After being dried naturally, they were fixed with neutral resin. The cells were evaluated under light microscopy, and the samples were classified as 1 of the 4 stages of the estrous cycle [16]. Diestrus vaginal smears were determined by the presence of high numbers of leukocytes; during proestrus, small-nucleated epithelial cells were present; in estrus, large numbers of cornified epithelial cells were observed; and in metestrus, leukocytes were also present.

### Morphological Observation

Pieces of ovary tissues (0.5 cm^3^ for each) were collected from the left or right ovary from each rat after euthanasia. Ovary tissues were fixed in 10% neutral-buffered formalin, embedded in paraffin, sectioned at 4 µm/slide, and stained with hematoxylin and eosin, dehydrated in 95, 90 and 70% ethanol, cleared in xylene. The sections were viewed using an Olympus DP73 microscope (Olympus, Tokyo, Japan).

### Transmission Electron Microscopy

The rat ovary tissues were cut into 1 mm^3^ strips, fixed for 4 h at 4°C in 5% glutaraldehyde, fully washed 3 times in 0.1 mol/L of PBS, post-fixed for 2 h at 4°C in 2% osmium tetroxide, and finally dehydrated in a graded series of ethanol baths. The samples were embedded in Epon 812, cut into ultrathin sections (75 nm) and stained with uranyl acetate and lead citrate. The sections were viewed using a HITACHI H-600 electron microscope at 80 kV (HITACHI, Tokyo, Japan).

### Enzyme Linked Immunosorbent Assay

Serum was extracted with diethyl ether, and the concentrations of T, ASD, E2, E1, P450arom, and P450c17α in the serum extracts were quantified using ELISA kits (Beinglay Biotechnology Co., Ltd., Wuhan, China).

### Immunohistochemical Assay

Sections were deparaffinized, hydrated, and then pretreated in a microwave (antigen retrieval). Endogenous peroxidase activity was inhibited with 0.3% H2O2, and nonspecific binding was blocked with 20% normal goat serum. All sections were incubated with P450arom (rabbit polyclonal anti-rat P450arom 1∶50, Thermo Fisher Scientific, Rockford, IL, USA) and P450c17α (rabbit monoclonal anti-rat P450c17α 1∶50, Abcam, Cambridge, UK) antibodies; for 2 h at 37°C. The samples were washed and then incubated for 30 min at room temperature with appropriate preabsorbed biotinylated secondary antibodies. The visualization of antigens was achieved using the streptavidin-peroxidase method (JRDUN Biotechnology Co., Ltd., Shanghai, China), and 3.3-diaminobenzidine (DAB) (Liquid DAB-Plus Substrate Kit, JRDUN Biotechnology Co., Ltd., Shanghai, China) was used as a chromogen. The slides were washed in distilled water and counterstained with Mayer’s hematoxylin; then, they were dehydrated and mounted. Antibodies were replaced with PBS for a negative control. For evaluation, a semi-quantitative analysis of the staining results was achieved using the IMS medical image quantitative analysis system (JRDUN Biotechnology Co., Ltd., Shanghai, China). Positive results for P450arom were brown or yellow particles stained among granular cells, while positive results for P450c17α were brown or yellow particles stained among theca cells. The positive area and the optical density (OD) values in 3 high-power optical fields (×200) of every slice were measured. The immune positive area index (positive area/total area ×OD) values of P450arom and P450c17α were calculated in every high-power optical field.

### Western Blotting

Each group of ovaries from the rats was homogenized in ice-cold lysis buffer (1.0 m Tris-Cl [pH 8.8], 150 mm NaCl, 1% Nonidet P-40, 0.5% sodium deoxycholate, 10% sodium dodecyl sulfate [SDS], 40 µm phenylmethylsulfonyl fluoride, 0.3 µm aprotinin, and 1 µm leupeptin). This step was followed by 30 min of incubation on ice and centrifugation at 12,000×g for 15 min at 4°C. The supernatant was transferred to new tubes and was aliquoted and stored at −80°C until electrophoresis. An aliquot of the supernatant was kept for protein measurement, using BSA as a standard. The samples were denatured by adding sample buffer (1.0 m Tris-HCl [pH 6.8], 2% SDS, 10% glycerol, 0.01% bromophenol blue), followed by boiling for 10 min. Then, 30 µg of protein was separated on 10% SDS-PAGE gels in Tris-glycine and 0.1% SDS buffer and transferred to nitrocellulose paper in 25 mm Tris, 192 mm glycine, and 20% methanol buffer at 250 mA for 1.5 h. The blots were incubated for 2 h at RT in 5% nonfat dry milk to block unspecific binding. The blots were then washed and incubated overnight at 4°C with anti-P450arom antibody (1∶500, Abcam, Cambridge, UK) and anti-P450c17 antibody (1∶400, Abcam, Cambridge, UK) at a 1/4000 dilution; the blots were then washed and incubated with a secondary antibody conjugated to horseradish peroxidase (1/6000 dilution) for 2 h at room temperature. Protein-antibody complexes were visualized using Western blotting luminol reagent following the manufacturer’s protocol (Abcam, Cambridge, UK).

### Real-time PCR

Total cellular RNA was extracted from the rat ovary tissue samples with RNA-Bee (Tel-Test, Friendswood, TX, USA). Three micrograms of total RNA was used as a template for reverse transcription using a Superscript Reverse Transcriptase kit (Invitrogen, Carlsbad, CA, USA). The cDNA samples were then submitted to PCR with the following primer pairs: for GAPDH, sense 5′-CCGAGGGCCCACTAAAGG-3′ and antisense 5′-GCTGTTGAAGTCACAGGAGACAA-3′; for P450arom sense, 5′ CTGGCTACTGTCTGGGAATC 3′ and antisense 5′ TTGCTGCCGAATCTGGAG 3′; and for P450c17α, sense 5′ CGAGAAGTGCTGCGTATC 3′ and antisense 5′ CTGCGTGGGTGTAATGAG 3′. Real time PCR was performed with a QuantiTect SYBR green PCR kit (ABI, USA) using an ABI 7500 real-time PCR system and SDS software (Applied Biosystems, Foster City, CA, USA). Data on mRNA expression are shown as the relative amount normalized to that of GAPDH.

### Statistical Analysis

The experimental data were shown as the means ± standard error of measurement ([SEM] ± S), and the SPSS statistical software package (SPSS, version16.0; Chicago, IL) was used for the statistical analysis. Multiple comparisons were performed using a one-way analysis of variance (ANOVA), followed by the correction of p values with Dunnett’s post hoc test; p<0.05 was set as the limit of statistical significance.

## Results

### Improvement in Estrous Cyclicity

**Figure 1:**
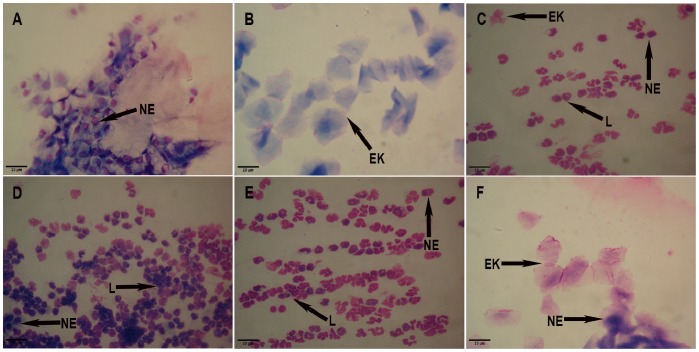
The vaginal smears of rats in the control, PCOS, and PCOS EA groups. Rats’ vaginal smears were stained with Wright’s stain, as described in the Materials and Methods section. A) The representative rat’s vaginal smears from the control group in proestrus (A×200). B) The representative rat’s vaginal smears from the control group in estrus (B×200). C) The representative rat’s vaginal smears from the control group in metestrus (C×200). D) The representative rat’s vaginal smears from the control group in diestrus (D×200). E) The representative rat’s vaginal smears from the PCOS group predominantly exhibited leukocytes, the main cell type observed during the diestrus stage (E×200). F) The representative rat’s vaginal smears from the PCOS EA group exhibited epithelial keratinocytes, the main cell type observed during the estrus stage (F×200). NE: nuclear epithelial cell, EK: epithelial keratinocyte, L: leukocyte. Scale bar, 20 µm.

The following items were detected under a microscope. The 10 rats in the control group had 4–5-day estrous cycles, comprising proestrus, estrus, metestrus and diestrus. In proestrus (Fig. 1A), oval nucleated epithelial cells, occasionally with a small number of keratinocytes, were detected. In estrus (Fig. 1B), epithelial keratinocytes with irregular shapes were detected; they resembled deciduous leaves or were interconnected into pieces, among which there was a small number of nuclear epithelial cells. In metestrus (Fig. 1C), irregular epithelial keratinocytes, nucleated epithelial cells, and leukocytes were detected. In diestrus (Fig. 1D), a large number of leukocytes and a small number of nuclear epithelial cells were detected .6 of 10 rats in the control group were in proestrus, and the remaining 4 rats were in estrus at the time of sacrifice.

The 10 rats in the PCOS group experienced prolonged diestrus beginning on the 7th day after the letrozole gavage, with estrus disappearing on approximately the 14th day and a large number of leukocytes and a small number of nuclear epithelial cells detected microscopically in the vaginal smears beginning on the 21st day (Fig. 1E). On the 10th day after the 10 rats in the PCOS group were treated with EA, epithelial keratinocytes were observed microscopically during estrus in the vaginal smears of 2 rats; on the 14th day of the EA treatment, epithelial keratinocytes were observed microscopically during estrus in the vaginal smears of 6 rats (Fig. 1F); the remaining 2 rats in the PCOS EA group were non responders to the EA treatment. In the PCOS EA group, 2 of 10 rats were in estrus, 6 of 10 rats were in metestrus, and the remaining 2 rats were in diestrus at the time of sacrifice. The 10 rats in the PCOS group were still in diestrus at the time of sacrifice.

### Ovarian Morphological Changes

**Figure 2:**
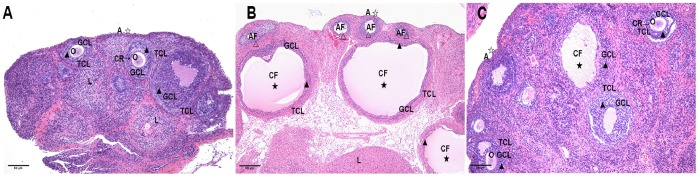
The morphological changes of the rats’ ovarian tissues in the control, PCOS, and PCOS EA groups. The morphological changes of the rats’ ovarian tissues were stained with hematoxylin and eosin, as described in the Materials and Methods section. A) A representative rat’s ovarian tissue section from the control group, which had normal appearance (A×100). B) A representative rat’s ovarian tissue section from the PCOS group showed thickening surface albuginea, under which there were many follicles in different phases (including atretic follicles and cystic dilating follicles), as well as fewer layers of granular cells, disappeared oocytes and corona radiating within the follicles (B×100). C) A representative rat’s ovarian tissue section from the PCOS EA group, which showed preantral and antral follicles, increased granular cell layers, and some ovulation phenomena (C×100). A: albuginea, AF: atretic follicle, CF: cystic follicle, CR: corona radiate, GCL: granular cell layer, L: luteal, O: oocyte, TCL: theca cell layer. ☆ indicated the changes of albuginea, ★ indicated the appearances of cystic follicle, △ indicated the appearances of atretic follicle, ▴ indicated the changes of granular cells layer. Scale bar, 50 µm.

Under light microscopy, the rat ovaries in the control group demonstrated multiple luteal, preantral, and antral follicles. The granular cells within the follicles showed multiple layers (8∼9 layers). Oocytes and corona radiata were visible in the follicles, part of which had been discharged (Fig. 2A). The rat ovaries in the PCOS group showed thickening surface albuginea, under which there were many follicles in different phases, including atretic follicles and cystic dilating follicles, as well as fewer layers of granular cells and disappeared oocytes and corona radiating within the follicles (Fig. 2B). The rat ovaries in the PCOS EA group showed preantral and antral follicles, increased granular cell layers (5∼8 layers), and some ovulation phenomena (Fig. 2C).

### Ovarian Ultrastructural Changes

**Figure 3:**
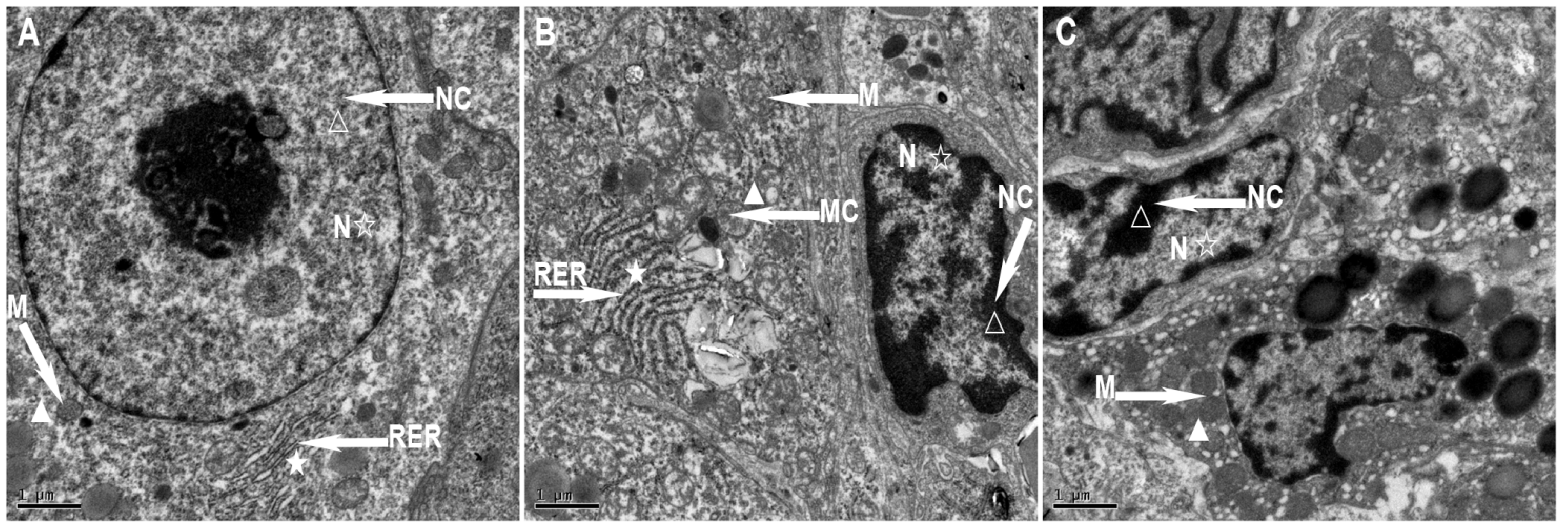
The ultrastructural changes of the rats’ ovarian tissues in the control, PCOS, and PCOS EA groups. The ultrastructural changes of the rats’ ovarian tissues were observed by transmission electron microscopy, as described in the Materials and Methods section. A) A representative rat’s ovarian tissue section from the control group, which had normal appearance (A×10000). B) A representative rat’s ovarian tissue section from the PCOS group, which showed a reduced number of organelles, condensation and margination of the nuclear chromatin, mitochondrial swelling, broken or even disappeared mitochondrial cristae, and expanded endoplasmic reticulum (B×10000). C) A representative rat’s ovarian tissue section from the PCOS EA group, which showed increased mitochondria in granular cells, many cristae (with some destroyed) in part of the mitochondria, condensation and margination of nuclear chromatin (C×10000). M: mitochondria, N: nucleus, RER: rough endoplasmic reticulum, NC: nuclear chromatin, MC: mitochondrial cristae. ☆ indicated the changes of nucleus, ★ indicated the changes of rough endoplasmic reticulum, △: indicated the changes of nuclear chromatin, ▴ indicated the changes of mitochondria and mitochondrial cristae. Scale bar, 1 µm.

Under the electron microscope, the rat ovaries in the control group demonstrated oval-shaped granular cells and basically normal nuclear morphology, with no expansion into the perinuclear space and continuous structure of the double nuclear membranes. In addition, there were basically normal mitochondria within the cytoplasm, evenly distributed chromatin in the nuclei, long oval mitochondria in the cytoplasm, many cristae in the mitochondria, a moderate amount of smooth endoplasmic reticulum and lipid droplets in the vacuoles, and a small amount of rough endoplasmic reticulum and Golgi (Fig. 3A). Under the electron microscope, the rat ovaries in the PCOS group showed deformed granular cells, a reduced number of organelles, a slightly incomplete karyotype, condensation and margination of the nuclear chromatin, mitochondrial swelling, broken or even disappeared mitochondrial cristae, and expanded endoplasmic reticulum (Fig. 3B). Under the electron microscope, the rat ovaries in the PCOS EA group showed increased mitochondria in granular cells, many cristae (with some destroyed) in part of the mitochondria, smooth endoplasmic reticula, Golgi complexes, ribosomes, a slightly incomplete karyotype, and condensation and margination of nuclear chromatin (Fig. 3C).

### Testosterone, Androstenedione, Estradiol, and Estrone Levels in the Rats’ Peripheral Sera (Fig. 4)

#### Comparison of the testosterone level in the rats’ peripheral sera from the different groups (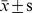)

**Figure 4:**
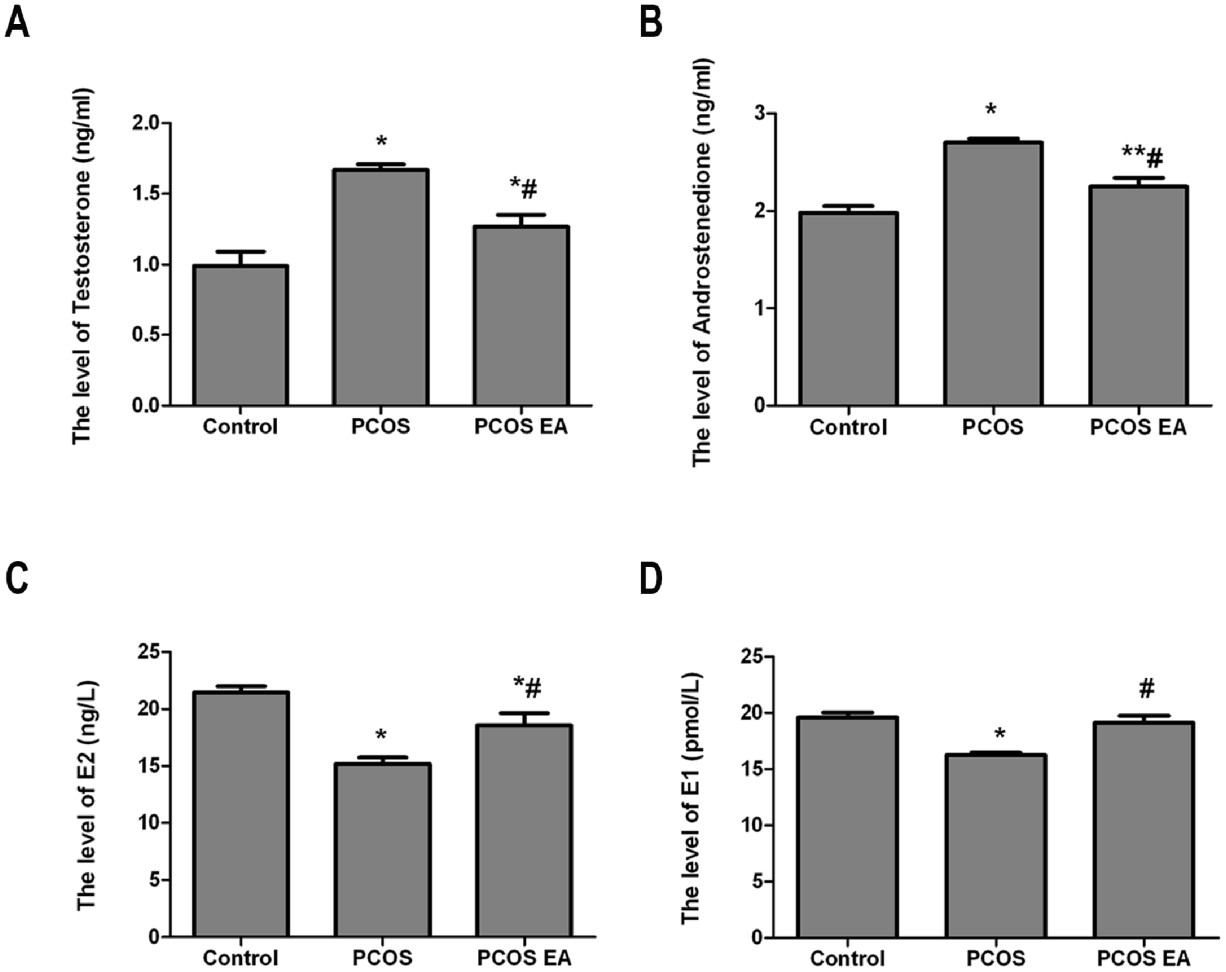
Comparisons of the testosterone, androstenedione, estradiol, and estrone levels in the rats’ peripheral sera from the control, PCOS, and PCOS EA groups (n = 10 rats/group). The levels of testosterone (A), androstenedione (B), estradiol (C), and estrone (D) in the sera extracts were quantified with ELISA kits, as described in the Materials and Methods section. The values were shown as the means ± SEMs. *p<0.01 vs. control, **p<0.05 vs. control, and ^#^p<0.01 vs. PCOS.

Compared with the control group, the rats from the PCOS and PCOS EA groups had significantly increased testosterone levels in the peripheral sera (p<0.01, p<0.01), while the rats in the PCOS EA group had significantly lower testosterone levels in their peripheral sera compared to the rats in the PCOS group (p<0.01) (Fig. 4A).

#### Comparison of the androstenedione level in the rats’ peripheral sera from the different groups (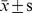)

Compared with the control group, the rats from the PCOS group had significantly increased androstenedione levels in their peripheral sera (p<0.01). Compared with the PCOS group, the rats in the PCOS EA group had significantly decreased androstenedione levels in their peripheral sera (p<0.01); however, the difference was still statistically significant compared with the control group (p<0.05) (Fig. 4B).

#### Comparison of the estradiol level in the rats’ peripheral sera from the different groups (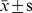)

Compared with the control group, the rats in the PCOS group had significantly decreased estradiol levels in their peripheral sera (p<0.01). Compared with the PCOS group, the rats in the PCOS EA group had significantly increased estradiol levels in their peripheral sera (p<0.01); however, the difference was still statistically significant compared with the control group (p<0.01) (Fig. 4C).

#### Comparison of the estrone level in the rats’ peripheral sera from the different groups (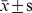)

Compared with the control group, the rats in the PCOS group had significantly decreased estrone levels in their peripheral sera (p<0.01). Compared with the PCOS group, the rats from the PCOS EA group had significantly increased estrone levels in their peripheral sera (p<0.01), with no significant difference compared to the control group (p>0.05) (Fig. 4D).

### P450arom and P450c17α Levels in the Rats’ Peripheral Sera (Fig. 5)

#### Comparison of the P450arom level in the rats’ peripheral sera from the different groups (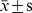)

**Figure 5:**
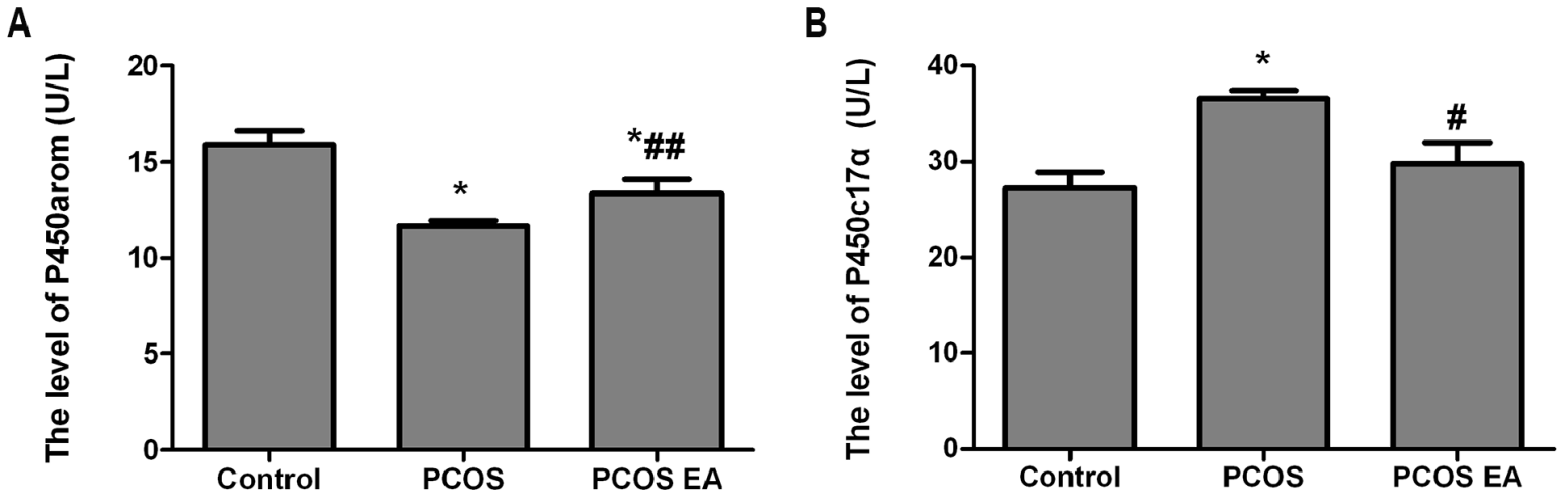
Comparisons of the P450arom and P450c17α levels in the rats’ peripheral sera from the control, PCOS, and PCOS EA groups (n = 10 rats/group). The levels of P450arom (A) and P450c17α (B) in the sera extracts were quantified with ELISA kits, as described in the Materials and Methods section. The values were shown as the means ± SEMs. *p<0.01 vs. control, ^#^p<0.01 vs. PCOS, and ^##^p<0.05 vs. PCOS.

Compared with the control group, the rats in the PCOS group had significantly decreased P450arom levels in their peripheral sera (p<0.01). Compared with the PCOS group, the rats in the PCOS EA group had significantly increased P450arom levels in their peripheral sera (p<0.05), and the difference was also significant compared with the control group (p<0.01) (Fig. 5A).

#### Comparison of the P450c17α level in the rats’ peripheral sera from the different groups (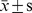)

Compared with the control group, the rats in the PCOS group had significantly increased P450c17α levels in their peripheral sera (p<0.01). Compared with the PCOS group, the rats in the PCOS EA group had significantly decreased P450c17α levels in their peripheral sera (p<0.01), with no significant difference compared with the control group (p>0.05) (Fig. 5B).

### P450arom and P450c17α Expressions in the Rats’ Ovarian Tissues (Fig. 6∼7)

#### Comparison of P450arom expression in the rats’ ovarian tissues from the different groups with immunohistochemistry (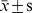, × 200) (Fig. 6)

**Figure 6:**
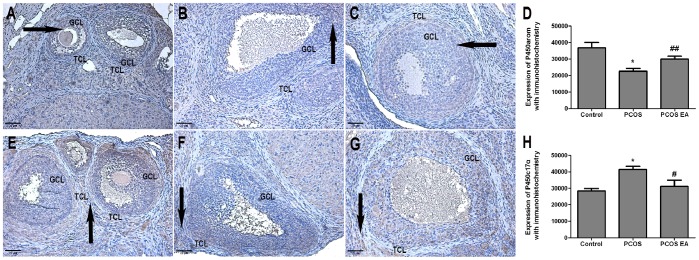
P450arom and P450c17α expressions in the rats’ ovarian tissues from the control, PCOS, and PCOS EA groups (n = 10 rats/group). The expressions of P450arom and P450c17α were assessed with immunohistochemistry, as described in the Materials and Methods section. A) The expression of P450arom in the rat’s ovarian tissue from the control group(A×200). B) The expression of P450arom in the rat’s ovarian tissue from the PCOS group (B×200). C) The expression of P450arom in the rat’s ovarian tissue from the PCOS EA group (C×200). D) Bar graph showed the expression of P450arom in the rats’ ovarian tissues from the control, PCOS and PCOS EA groups. E) The expression of P450c17α in the rat’s ovarian tissue from the control group (E×200). F) The expression of P450c17α in the rat’s ovarian tissue from the PCOS group (F×200). G) The expression of P450c17α in the rat’s ovarian tissue from the PCOS EA group (G×200). H) Bar graph showed the expression of P450c17α in the rats’ ovarian tissues from the control, PCOS and PCOS EA groups. The values were shown as the means ± SEMs. *p<0.01 vs. control, ^#^p<0.01 vs. PCOS, and ^##^p<0.05 vs. PCOS. Arrows in A to C indicated the positive expressions of rat ovarian granulosa cell layer. Arrows in E to G indicated the positive expressions of rat ovarian theca cell layer. GCL: granular cell layer, TCL: theca cell layer. Scale bar, 20 µm.

P450arom is mainly expressed in rat ovarian granulosa cell layer (Fig. 6A–C). Compared with the control group, the rats from the PCOS group had significantly decreased P450arom expression in the ovarian tissues (p<0.01) (Fig. 6A, 6B, 6D). Compared with the PCOS group, the rats from the PCOS EA group had significantly increased P450arom expression in their ovarian tissues (p<0.05) (Fig. 6B, 6C, 6D), with no significant difference compared with the control group (p>0.05) (Fig. 6A, 6C, 6D).

#### Comparison of P450c17α expression in the rats’ ovarian tissues from the different groups with immunohistochemistry (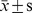, ×200) (Fig. 6)

P450c17α is mainly expressed in rat ovarian theca cell layer (Fig. 6E–G). Compared with the control group, the rats from the PCOS group had significantly increased P450c17α expression in the ovarian tissues (p<0.01) (Fig. 6E, 6F, 6H). Compared with the PCOS group, the rats from the PCOS EA group had significantly decreased P450c17α expression in their ovarian tissues (p<0.01) (Fig. 6F, 6G, 6H), with no significant difference compared with the control group (p>0.05) (Fig. 6E, 6G, 6H).

#### Comparison of P450arom expression in the rats’ ovarian tissues from the different groups with Western blotting analysis (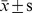) (Fig. 7)

**Figure 7:**
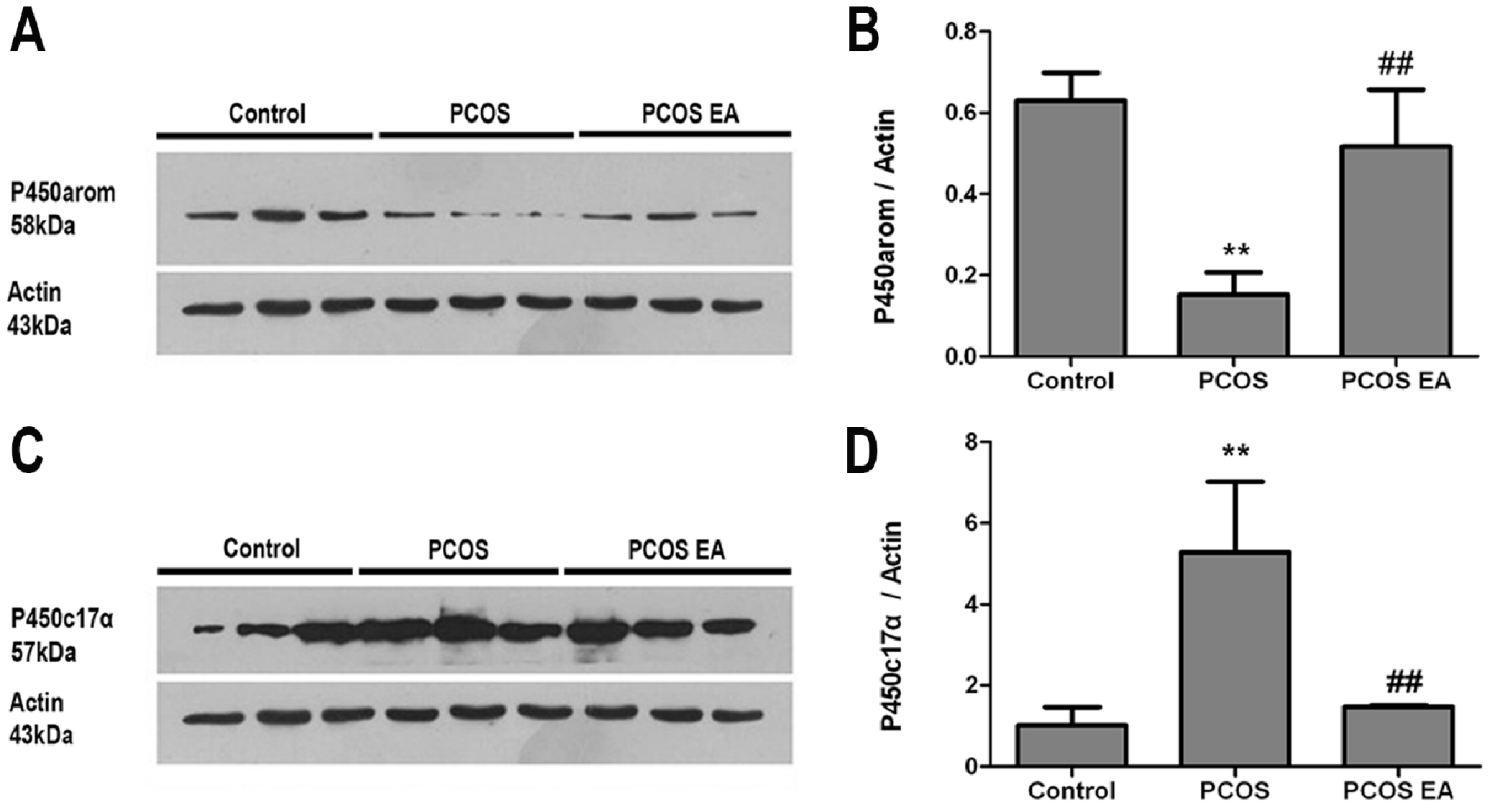
P450arom and P450c17α expressions in the rats’ ovarian tissues from the control, PCOS, and PCOS EA groups (n = 3 rats/group). The expressions of P450arom and P450c17α were assessed with Western blotting analysis, as described in the Materials and Methods section. A) Representative Western blot of P450arom and actin. B) Bar graph showed the results, which were expressed as ratio of P450arom to that of the actin bands in the rats’ ovarian tissues from the control, PCOS and PCOS EA groups. The values were shown as the means ± SEMs. **p<0.05 vs. control group, ^##^p<0.05 vs. PCOS group. C) Representative Western blot of P450c17α and actin. D) Bar graph showed the results, which were expressed as ratio of P450c17α to that of the actin bands in the rats’ ovarian tissues from the control, PCOS and PCOS EA groups. The values were shown as the means ± SEMs. **p<0.05 vs. control group, ^##^p<0.05 vs. PCOS group.

Compared with the control group, the rats from the PCOS group had significantly decreased P450arom expression in their ovarian tissues (p<0.05). Compared with the PCOS group, the rats from the PCOS EA group had significantly increased P450arom expression in their ovarian tissues (p<0.05), with no significant difference compared with the control group (p>0.05) (Fig. 7A, 7B).

#### Comparison of P450c17α expression in the rats’ ovarian tissues from the different groups with Western blotting analysis (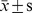) (Fig. 7)

Compared with the control group, the rats from the PCOS group had significantly increased P450c17α expression in their ovarian tissues (p<0.05); compared with the PCOS group, rats from the PCOS EA group had significantly decreased P450c17α expression in their ovarian tissues (p<0.05), with no significant difference compared with the control group (p>0.05) (Fig. 7C, 7D).

### P450arom mRNA and P450c17α mRNA Expressions in the Rats’ Ovarian Tissues (Fig. 8)

#### Comparison of P450arom mRNA expression in the rats’ovarian tissues from the different groups (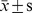)

**Figure 8:**
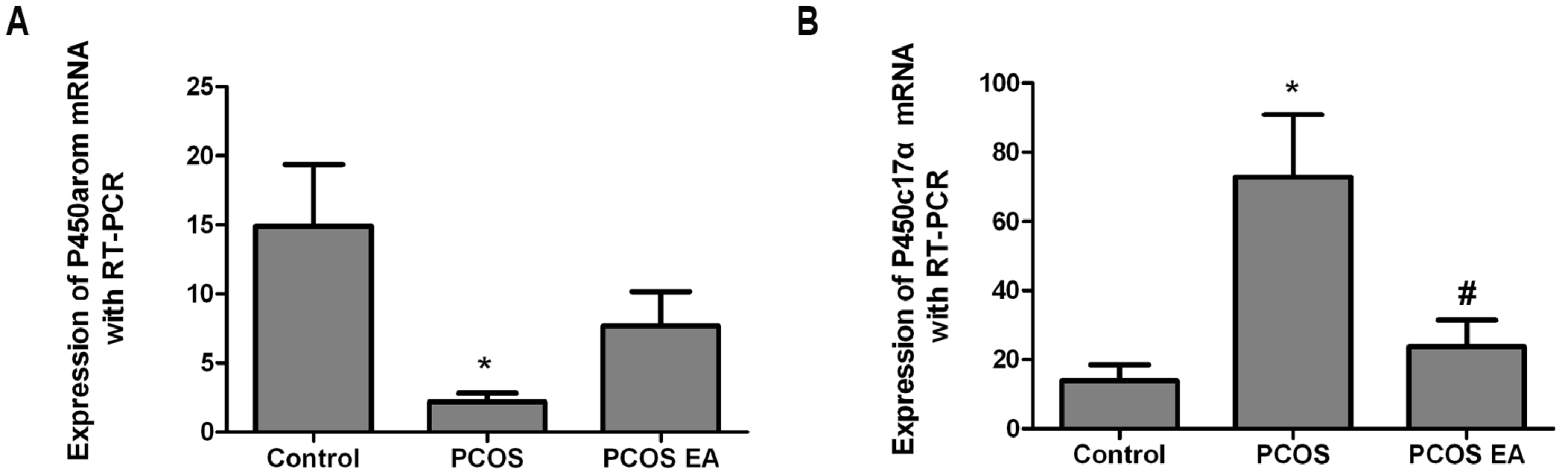
P450arom mRNA and P450c17α mRNA expressions in the rats’ ovarian tissues from the control, PCOS, and PCOS EA groups (n = 10 rats/group). The expressions of P450arom mRNA (A) and P450c17α mRNA (B) were measured with real-time PCR, as described in the Materials and Methods section. The values were shown as the means ± SEMs. *p<0.01 vs. control, ^#^p<0.01 vs. PCOS.

Compared with the control group, rats from the PCOS group had significantly decreased P450arom mRNA expression in their ovarian tissues (p<0.01). Compared with the PCOS group, rats from the PCOS EA group had some increased P450arom mRNA expression in their ovarian tissues, but with no significant difference (p>0.05) (Fig. 8A).

#### Comparison of P450c17α mRNA expression in the rats’ovarian tissues from the different groups (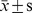)

Compared with the control group, the rats from the PCOS group had significantly increased P450c17α mRNA expression in their ovarian tissues (p<0.01). Compared with the PCOS group, the rats from the PCOS EA group had significantly decreased P450c17α mRNA expression in their ovarian tissues (p<0.01), with no significant difference with the control group (p>0.05) (Fig. 8B).

## Discussion

PCOS is difficult to treat because of its complex etiology and pathogenesis as well as its heterogeneous or polymorphic clinical manifestations. The treatment effects suggest that regulating abnormal androgen and/or estrogen is an effective means of treating PCOS. In recent years, clinical studies have demonstrated the effectiveness of EA treatment for PCOS [11]–[14]. The mechanism of EA treatment for PCOS has been gradually revealed. Stener-Victorin et al. found that low-frequency EA stimulation reduced estradiol valerate-induced ovarian nerve growth factor (NGF) [5], ovarian corticotropin-releasing factor (CRF) [6], and ovarian and hypothalamic endothelin-1 (ET-1) levels [7]. In addition, the following observations were made in that study: EA weakened the PCOS rats’ ovarian NGF mRNA and ovarian adrenaline receptor β (2)-ARs mRNA expression and decreased the protein contents of the adrenergic receptors α (1)a-Ars and α (1)d-ARs and the low-affinity neurotrophin receptor P75NTR [9]; EA improved local ovarian blood flow [17]–[18] and ovarian tissue morphology [19]; EA reduced PCOS rats’ GnRH-immunoreactive cell expression in the medial preoptic area (MPO) and in the nucleus of the diagonal band for HDB; and EA weakened the protein expression of hypothalamic androgen receptor (AR) [20]. The Stener-Victorin et al. studies demonstrated that EA exhibits neuroendocrine effects on PCOS at the peripheral (ovarian) and central (hypothalamus) levels.

Local excessive ovarian androgen, or hyperandrogenism, and anovulation are typical clinical features of PCOS [21]. As a physiological regulator of neuroendocrine mechanisms, androgen can affect cyclical ovulation [22]–[26]. The local and peripheral blood circulation of PCOS patients’ ovaries often contains excessive androgen, which can cause early luteinization of ovarian granular layer cells, stopping follicular development and growth and leading to follicle atresia (caused by the lack of a dominant follicle) and eventually anovulation or poor ovulation.

Recent studies have found that P450c17α and P450arom, as 2 key enzymes of the synthesis or catalysis of androgen and estrogen in ovarian tissues, play important roles in maintaining the environmental balance of local ovarian hormones [3]–[4]. P450c17α expression mainly occurs in the ovarian thecal cell layer for the catalysis of the conversion of pregnenolone into 17α-hydroxy pregnenolone and DHEA as well as the conversion of progesterone into 17α-hydroxyprogesterone and androstenedione [27]–[29]. Clinical observation has shown increased luteinizing hormone levels in the peripheral blood of PCOS patients [30], acting directly on the luteinizing hormone receptor of theca cells [31], which activates P450C17α activity within theca cells, whereas as a key enzyme in androgen synthesis, P450c17α’s increased activity or expression can promote excessive androgen synthesis, leading to local excessive ovarian androgen or hyperandrogenism in PCOS patients. P450arom expression in the ovarian granular cell layer enables the conversion of testosterone and androstenedione into estradiol and estrone [3], [32], whose content and/or activity directly determine the levels of estrogen in the ovarian tissue. In addition, under the impact of follicle-stimulating hormone and its receptor of ovarian granular cells, P450arom promotes the proliferation and differentiation of granular cells, and it affects follicle advantage by affecting the connective tissue growth factor [33].

To determine whether P450 aromatase was a key enzyme for estrogen and for aromatase inhibitors blocking the conversion of androgen into estrogen, we prepared a PCOS research model with letrozole, a non-steroidal aromatase inhibitor. Observation showed that 14 days after the gavage of letrozole, the estrus cycle disappeared, while the diestrus cycle extended. In addition, 21 days after the gavage of letrozole or at the end of modeling, all rats were in diestrus. Vaginal smears showed a large number of white blood cells and a small number of nuclear epithelial cells. Under light microscopy, ovarian tissues showed obvious polycystic changes; capsular thickening; albuginea containing preantral, antral, and atresia; cystic dilating follicles; reduced granular cell layers; and disappeared oocytes and corona radiata in the follicles. Under an electron microscope, the tissues showed deformed granular cells, reduced organelles, mitochondrial swelling, broken or even disappeared mitochondrial cristae, and dilated endoplasmic reticulum, as well as decreased estrone and estradiol levels and significantly increased androstenedione and testosterone levels in the peripheral blood (concurrently). These findings suggest that the PCOS model, induced by aromatase inhibitor letrozole in our research, exhibited similar endocrine and histological changes to those in human PCOS, which agreed with the results of the azole-induced PCOS model by Kafali et al. [15].

In this research, we chose CV-4 and CV-3 for low-frequency 2 Hz EA stimulation in the PCOS rats. These 2 acupoints, located on the lower abdomen and at the same segmental area as the uterus and ovaries, are common sites for treating reproductive and endocrine diseases. After 14 consecutive days of EA treatment, the PCOS rats underwent significant changes in ovarian tissue morphology, and the theca cell layer P450C17α and its mRNA expression were weakened. There were also significant decreases in the testosterone, androstenedione, and P450C17α levels in the peripheral blood. Stener-Victorin et al. observed that low-frequency EA modulated the central β-endorphin system, exerting regulatory control on the GnRH pulse generator and on pituitary LH release in PCOS [34]–[35]. We observed a reduction in the theca cell layer P450C17α and its mRNA expression after low-frequency EA stimulation in the PCOS EA group. However, in-depth research is still required to determine whether low levels of LH, after inhibition of hypothalamic GnRH neuron pulse generators and pituitary LH release by EA, act on LH receptors in theca cells and reduce intracellular P450C17α and mRNA expression or if EA can directly regulate P450C17α and mRNA expression.

We also observed increased P450arom and its mRNA expression in granular cell layers as well as increased estradiol, estrone and P450arom levels in peripheral blood after low-frequency EA stimulation in PCOS rats. Okubo T et al. [36] observed P450arom expression in follicles before ovulation and in luteal cells in the ovaries of women of childbearing age. However, P450arom expression first occurred in ovarian granular cells under the regulation of follicle-stimulating hormone, and granular cells demonstrated the highest expression. Using laser microdissection, Yosuke Sakurada et al. [37] observed an increase in P450arom mRNA expression, along with the development of associated follicles in immature ovaries. Immunostaining for P450arom protein suggested that the granular cells of large antral follicles expressed more P450arom mRNA compared with preantral follicles. Araki et al. [38] observed that P450arom activity was highly expressed in granular cells of follicles ≥8 mm in diameter, and the expression increased as the follicle size increased. From these studies, we know that P450arom and its mRNA expression in ovarian granular cells are related to morphological changes in granular cells, which is the same conclusion that was reached in our research. Rats in the PCOS group had poorly developed primary follicles and cystic dilating follicles, as well as significantly reduced granular cell layers, whereas rats in the PCOS EA group had some matured antral follicles, hyperplasia, increased mitochondria in granular cells, and increased expression of P450arom and its mRNA, as well as increased estrone and estradiol levels. Cameron et al. [39] found that the estrogen levels in the rats’ peripheral sera were highest in proestrus while lowest in diestrus. The cycle phases of the rats from different groups at the time of sacrifice in our research were consistent with the estrogen levels. In addition, 8 of 10 rats in the PCOS EA group restored their estrous cycles with these changes in the ovaries, suggesting that ovulation phenomena occurred in these rats.

In conclusion, we observed that after 14 consecutive days of EA treatment, the PCOS rats gradually recovered from a “disappeared estrus, extended diestrus” state to an estrous cycle, and their ovarian tissue morphologies tended to normalize. Theca cell layer P450C17α and its mRNA expression weakened, while granular cell layer P450arom and its mRNA expression increased. Furthermore, androstenedione and testosterone, synthesized or catalyzed by P450C17α in the peripheral blood, significantly decreased, while estradiol and estrone, synthesized or catalyzed by P450arom, significantly increased. These results suggest that EA could impact P450arom and P450c17α as well as the expression of their mRNA in PCOS ovarian tissues, thereby changing the local ovarian environment of excessive androgen and improving the reproductive, endocrine, and metabolic disorders associated with PCOS.
